# Genetic Disruption of the Copulatory Plug in Mice Leads to Severely Reduced Fertility

**DOI:** 10.1371/journal.pgen.1003185

**Published:** 2013-01-17

**Authors:** Matthew D. Dean

**Affiliations:** Department of Molecular and Computational Biology, University of Southern California, Los Angeles, California, United States of America; University of Illinois, United States of America

## Abstract

Seminal fluid proteins affect fertility at multiple stages in reproduction. In many species, a male's ejaculate coagulates to form a copulatory plug. Although taxonomically widespread, the molecular details of plug formation remain poorly understood, limiting our ability to manipulate the structure and understand its role in reproduction. Here I show that male mice knockouts for *transglutaminase IV* (*Tgm4*) fail to form a copulatory plug, demonstrating that this gene is necessary for plug formation and lending a powerful new genetic tool to begin characterizing plug function. *Tgm4* knockout males show normal sperm count, sperm motility, and reproductive morphology. However, very little of their ejaculate migrates into the female's reproductive tract, suggesting the plug prevents ejaculate leakage. Poor ejaculate migration leads to a reduction in the proportion of oocytes fertilized. However, *Tgm4* knockout males fertilized between 3–11 oocytes, which should be adequate for a normal litter. Nevertheless, females mated to *Tgm4* knockout males for approximately 14 days were significantly less likely to give birth to a litter compared to females mated to wild-type males. Therefore, it appears that the plug also affects post-fertilization events such as implantation and/or gestation. This study shows that a gene influencing the viscosity of seminal fluid has a major influence on male fertility.

## Author Summary

Male reproductive fitness is strongly affected by seminal fluid. In many animals, the male's ejaculate coagulates in the female's reproductive tract to form a structure known as the copulatory plug. Here, I show that male mice without a functional copy of the gene *transglutaminase IV* cannot form a plug and suffer severe fertility defects. In spite of normal reproductive morphology, less of the ejaculate migrates through the female's reproductive tract and *Tgm4* knockout males sire significantly fewer litters than wild type. This study demonstrates that the copulatory plug and/or Tgm4 itself is necessary for normal fertility.

## Introduction

The non-sperm component of an ejaculate can have large effects on male reproductive fitness. In internally fertilizing species, seminal proteins can modify female receptivity [1]–[3], egg laying behavior [4]–[6], implantation and gestation [7], and the female's immune response to sperm and embryo [7]–[11]. Seminal proteins can also interact with the ejaculates of competitor males to influence the outcomes of fertilization [12]–[14]. In many internally fertilizing taxa, ejaculated proteins coagulate to form a hardened copulatory plug in the vaginal-cervical region of the female [15]–[22]. In spite of its wide taxonomic distribution, the molecular details that underlie its formation remain poorly understood, which limits investigations into its function. After reviewing previous biochemical insights, I present a new genetic model that offers unprecedented power to being dissecting the function of the plug.

Since the first published observation of a copulatory plug in a rodent nearly 165 years ago [19], several groups have attempted to characterize its molecular basis. Camus and Gley [23] showed that fluids extracted from the seminal vesicles coagulated *in vitro* upon contact with extract from the anterior lobe of the prostate (also referred to as the coagulating gland) [24], [25]. Building from the Camus & Gley experiment, Williams-Ashman and colleagues showed that the rate of coagulation depended on the concentrations of seminal vesicle and/or prostate protein extracts *in vitro* [26]. Because these early experiments were based on crude extracts, the general term “vesiculase” was coined to describe the unknown prostate-derived protein(s) responsible for inducing the coagulation of seminal vesicle proteins. More detailed biochemical investigations suggested the unknown vesiculase(s) was a transglutaminase [27], [28], a protein that crosslinks glutamines and lysines via γ-glutamyl-ε-lysine dipeptide bonds and causes the bound proteins to become insoluble and coagulate. A prostate-specific transglutaminase, *transglutaminase IV* (*Tgm4*), was later characterized from humans [29]–[31], and its protein is found in human ejaculates [32]. The ortholog in mouse is also ejaculated [33], and functionally analogous transglutaminases have been found in mosquito ejaculates [16].

In spite of these early advances, it remains unknown whether Tgm4 is necessary for the formation of the copulatory plug. It has been suggested that some seminal vesicle proteins self-coagulate in the absence of Tgm4 [34], that proteins other than Tgm4 induce the coagulation [35], and that female-derived proteins may be necessary for coagulation [36]. Furthermore, there is evidence that more than one transglutaminase exists in the male reproductive tract of rodents [33], [37], [38], though this could also be due to post-translational modifications [39]. Interestingly, human ejaculates do not coagulate strongly even though they have large amounts of Tgm4 [32], calling into question its role in seminal fluid coagulation.

More fully characterizing the biochemical basis of seminal fluid coagulation is critical for understanding the function of the copulatory plug. Early attempts to study copulatory plug function necessarily relied on surgical removal of male accessory glands [40]–[47]. Although copulatory plugs were abnormal and male fertility compromised in some cases, inferences were limited by the invasiveness of the procedures, the confounding effects associated with the potential alteration of hundreds of ejaculated proteins, and the failure to fully prevent a copulatory plug-like structure from forming. Other experiments showed that manual removal of the plug soon after copulation did not prevent pregnancy or parturition [48], [49]. However, the copulatory plug may have affected fertility prior to experimental removal. To address these early experimental limitations requires a method to fully prevent the formation of a copulatory plug with minimal invasiveness.

Here, I use *Tgm4* knockout mice to better understand the molecular basis and functional importance of the copulatory plug, and report two main findings. First, *Tgm4* knockout males failed to produce a copulatory plug after mating, demonstrating for the first time that this gene is necessary for the coagulation of seminal fluid in mice. *Tgm4* knockout males therefore provide a powerful model to investigate the function of the copulatory plug. Second, in spite of normal sperm count, sperm motility, and reproductive morphology, *Tgm4* knockout males sired significantly fewer litters than their wild type brothers. Analyses presented below suggest *Tgm4* knockout males suffer fertility defects at two important stages: 1) less of their ejaculate migrates into the female's reproductive tract, and 2) females mated to *Tgm4* knockout males produce significantly fewer litters even though a “normal” absolute number of oocytes were fertilized, suggesting additional defects in implantation and/or gestation. This study demonstrates that a gene influencing the viscosity of semen has major affects on male reproductive success.

## Results

Heterozygous “knockout first” mice were acquired from the Knockout Mouse Project (see [50],[51], and Materials and Methods). Heterozygotes were crossed in the laboratory to generate homozygous and heterozygous knockout males, as well as homozygous wild type males that were used as controls in all experiments. All females used throughout the manuscript were homozygous wild type. All mice were essentially genetically identical except for the ∼7 kb “knockout first” cassette that spans exons 2–3 of *Tgm4*.

*Tgm4* knockout males (homozygous for the “knockout first” allele) did not form a copulatory plug (Table 1), demonstrating for the first time that this gene is necessary for seminal fluid coagulation. From 13 successful 3-hour pairings to *Tgm4* knockout males (“success” being defined as the presence of sperm somewhere in the female's reproductive tract after three hours of pairing), complete dissection of each female's reproductive tract failed to yield a copulatory plug or plug-like structure (Table 1). In contrast, 14 of 16 successful 3-hour pairings to wild type males resulted in a copulatory plug, which normally occupies most of the vaginal canal and extends into the cervix, appearing “glued” to the epithelium. Herein, “wild type” includes males that were either heterozygous or homozygous for the wild type allele, as they were phenotypically indistinguishable from each other. I obtained similar results from 20-hour long male-female pairings: 0 of 8 females successfully paired with *Tgm4* knockout males, and 11 of 15 paired to wild type males, yielded a plug. Because they cannot form a plug, *Tgm4* knockout males represent a powerful genetic tool to investigate its role in reproduction.

**Table 1:** Results from 3 h and 20 h pairings of experimental males to homozygous wild type 6N females.

Cross duration	Male genotype^1^	Attempted^2^	Successful^3^	Copulatory plug^4^	Fertilized^5^	Not fertilized^6^
3 hours	KO	28	13 (46%)	0 (0%)	0 (0%)	216 (100%)
	+	26	16 (62%)	14 (88%)	0 (0%)	345 (100%)
20 hours	KO	27	8 (30%)	0 (0%)	45 (37%)	77 (63%)
	+	22	15 (68%)	11 (73%)	153 (66%)	78 (34%)

1 Females were paired with homozygous knockout (KO) or wild type (+, possessing at least one functional allele) males for 3 hours or 20 hours (see text).

2 Number of matings attempted.

3 Number (percentage) of pairings for which sperm were observed somewhere in the female reproductive tract.

4 Number (percentage) of successful crosses that resulted in a copulatory plug.

5 Number of fertilized and

6 unfertilized oocytes after 3 hours or 20 hours of pairing. Two pronuclei are only visible after 20 hours of crossing. When analyzed on a per-female basis (see text) *Tgm4* knockout males fertilized significantly fewer oocytes than wild type in the 20 hours crosses (*t* = 3.00, df = 15.14, P = 0.01).

**Figure 1:**
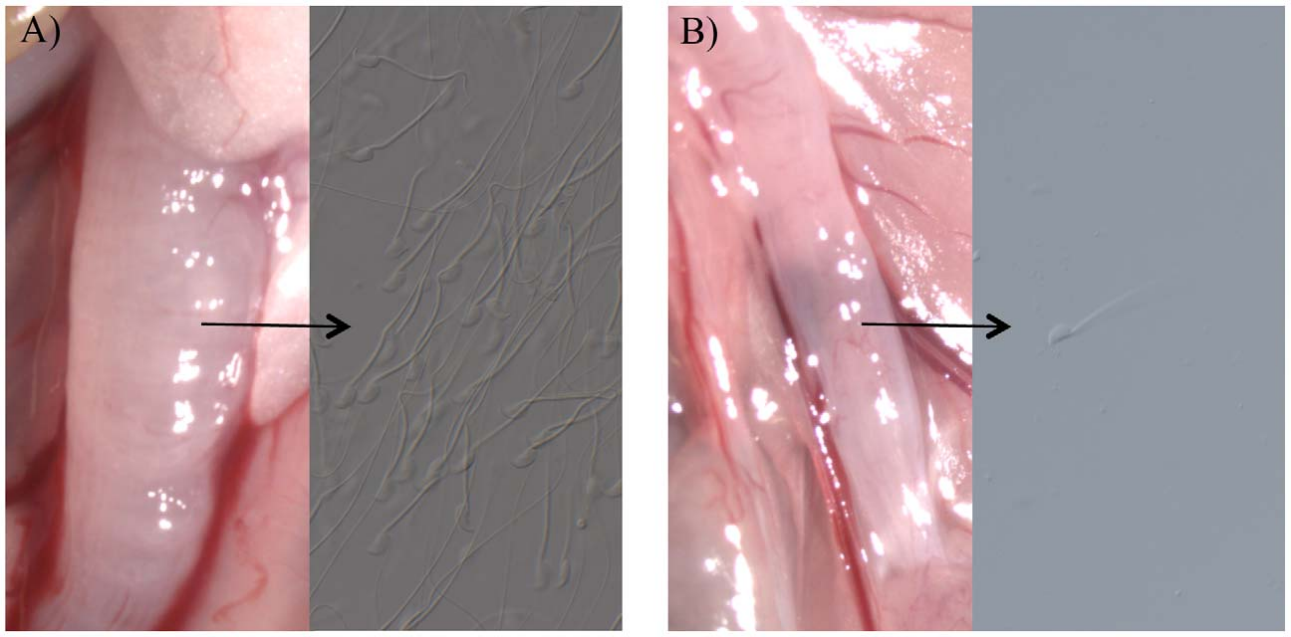
The appearance of female uterine horns after mating. The appearance of female uterine horns after mating to A) a wild type male, where uterine horns swelled with the male's ejaculate, or B) a *Tgm4* knockout male, where uterine horns qualitatively resembled unmated females, with no swelling. Very few sperm were observed in females mated to *Tgm4* knockout males.

In the absence of a plug, the ejaculates of *Tgm4* knockout males did not traverse the female reproductive tract properly. After mating to wild type males, female uterine horns appeared swollen, full of sperm and seminal fluid (Figure 1A). In contrast, after mating to *Tgm4* knockout males, female uterine horns did not swell and sperm were difficult to locate upon dissection (Figure 1B). The difference in uterine horn width was statistically significant between females mated to wild type (N = 6) vs. *Tgm4* knockout males (N = 15) (wild type: 2.64 mm, SD = 0.30; *Tgm4* knockout: 2.14 mm, SD = 0.43; *t* = 2.98, df = 19, P = 0.01).

The defect in ejaculate migration cannot be explained by defects in reproductive morphology of *Tgm4* knockout males. Sperm count was not statistically different between wild type (N = 19) vs. *Tgm4* knockout (N = 10) males (mean = 133,900 sperm/µl, SD = 55,000 vs. mean = 106,000 sperm/µl, SD = 43,000, respectively: *t* = 1.39, df = 27, P = 0.18), nor was sperm motility (mean = 0.96 sperm/sec, SD = 0.28 vs. mean = 0.87 sperm/sec, SD = 0.27, respectively: *t* = 0.78, df = 27, P = 0.44). From these same males, testis and seminal vesicle weight were analyzed in a full factorial ANCOVA to account for the potential covariation with body weight. There was not a significant difference in testis weight between *Tgm4* knockout vs. wild type males (F_1,25_ = 0.02, P = 0.88), nor was there a genotype×body weight interaction effect on testis weight (F_1, 25_ = 0.95, P = 0.34). Similarly, there was no difference in seminal vesicle weight between genotypes (F_1, 25_<0.01, P = 0.97), nor was there a genotype×body weight interaction effect on seminal vesicle weight (F_1, 25_ = 0.03, P = 0.86). Furthermore, *Tgm4* knockout males successfully copulated at a rate similar to wild type; from the 3-hour pairings, 16/26 to wild type, and 13/28 pairings to *Tgm4* knockout males succeeded (Table 1, χ^2^ = 0.7, P = 0.4). Therefore, both genotypes display normal copulatory behavior.

As might be expected from the reduced number of sperm that make it into the female's uterus, *Tgm4* knockout males fertilized significantly fewer oocytes in 20-hour assays. Among successful 20-hour pairings, *Tgm4* knockout males fertilized 45 of 122 oocytes dissected from the female's oviducts (36.9%), compared to wild type males, which fertilized 153 of 231 oocytes (66.2%) (Table 1). Fertilized oocytes from all successful pairings appeared healthy, with almost no signs of fragmentation. Oocytes originating from the same female are not independent observations, so I compared the proportion of fertilized oocytes on a per-female basis. Seven females successfully mated to *Tgm4* knockout males yielded a mean 39.4% fertilized oocytes (range 21.1%–72.7%), significantly lower than 12 females successfully mated to wild type (mean = 67.9%, range 12.5%–93.3%) (*t* = 2.78, df = 17, P = 0.01). The number of females analyzed (7 mated to *Tgm4* knockout and 12 mated to wild type) does not add up to the numbers in Table 1 (8 mated to *Tgm4* knockout and 15 mated to wild type) because scorable oocytes were not always recovered from oviduct dissections. It should be noted that even though *Tgm4* knockout males fertilized a lower *proportion* of oocytes compared to wild type, they always fertilized at least 3 oocytes (mean = 6.4, range 3–11), suggesting they should be able to impregnate females without difficulty.

In contrast to this prediction, after being paired with females for 10–14 days, *Tgm4* knockout males sired significantly fewer litters than wild type (Table 2). Of 30 pairings with *Tgm4* knockout males, only 17 produced litters, significantly fewer than wild type males, which produced litters in 135 of 165 pairings (χ^2^ = 7.93, P = 0.005) (Table 2). Among all litters born to wild type fathers, 42/106 (39.6%) yielded 6 or fewer pups (the mean number of oocytes fertilized by *Tgm4* knockout males, see previous paragraph), and 14/106 (13.2%) resulted in 3 or fewer pups (the minimum number of oocytes fertilized by *Tgm4* knockout males, see previous paragraph). In other words, *Tgm4* knockout males sired significantly fewer litters in spite of the fact that they appeared to fertilize enough oocytes for a healthy litter.

**Table 2:** Results of pairing experimental males to homozygous wild type 6N females for 10–14 days.

Male Genotype^1^	Attempted^2^	Litters born^3^	Litters weaned^4^
KO	30	17 (57%)	11 (37%)
+	165	135 (82%)	106 (64%)

1 Females paired with *Tgm4* knockout males (KO) gave birth to litters significantly less frequently than when paired with males carrying at least one functional Tgm4 copy (+). (χ2 = 7.934, P = 0.005).

2 The number of male and females paired for 10–14 days. To control for maternal experience, any pairings that occurred prior to a female's first successful litter were excluded.

3 Of attempted pairings, the number that resulted in a litter born.

4 Of attempted pairings, the number that resulted in a weaned litter 21–28 days after birth. Of litters born, there was no significant difference in the number of litters that reached weaning (χ2 = 0.94, P = 0.3).

Although *Tgm4* knockout males sired significantly fewer litters (Table 2), there were no signs of maternal neglect, as judged by the likelihood a litter reached weaning age, the litter size, and the size of offspring at weaning. Specifically, 11 of 17 (65%) litters sired by *Tgm4* knockout males reached weaning age (21–28 days old), compared to 106 of 135 (79%) litters born to a wild type male (Table 2; χ^2^ = 0.94, P = 0.3). Sometimes litters do not reach weaning age because of maternal neglect. Furthermore, the number of offspring weaned per litter was not significantly different among the two male genotypes (mean = 6.0 vs. 6.4 pups weaned per litter, SD = 3.5 vs. 2.4, range 1–12 for both, from N = 11 vs. 106 weaned litters sired by *Tgm4* knockout or wild type males, respectively: Welch's *t* = 0.59, df = 10.97, P = 0.57), nor was weanling weight (mean weight = 11.61 g vs. 12.14 g, SD = 3.0 vs. 3.8 from N = 66 pups weighed from 11 litters vs. 77 pups weighed from 13 litters sired by *Tgm4* knockout or wild type males, respectively: Welch's *t* = 0.93, df = 140.0, P = 0.35). The lack of statistical significance may be due to small sample sizes, but suggests that once litters are born, the pups have an equal chance of reaching healthy weaning age regardless of sire genotype.

## Discussion

*Tgm4* knockout males failed to produce a copulatory plug, demonstrating for the first time that this gene is necessary for plug formation. In spite of normal sperm count, motility and reproductive morphology, *Tgm4* knockout males suffered reduced fertility, most importantly in the significant reduction of litters born compared to wild type. Taking all the data into consideration, a model of the copulatory plug acting at two important stages of reproduction seems to explain the fertility defects of *Tgm4* knockout males. First, the plug may facilitate passage of the ejaculate through the cervix and into the uterine horns and oviducts (Fig. 1, Table 1), perhaps by sealing off the vagina and preventing backflow of the ejaculate [16], [41], [42], [52]–[54]. Second, the plug may enhance the embryos' ability to implant in the female's uterus, and/or reduce the chances of abortion after implantation (Table 2). For example, the plug may contribute to the physical stimulation necessary to shift the female's physiology towards “pseudopregnancy” [53], [55]–[57], a state where the uterus becomes primed for implantation in mice. This second aspect of the model is supported by the reduced number of litters born to *Tgm4* knockout males in spite of the fact that they fertilized between 3–11 oocytes in 20-hour assays. There does not appear to be any fertility defects that arise from differential maternal investment post-parturition.

Four observations suggest that the fertility defects observed in the current study arose from the absence of the copulatory plug rather than from additional pleiotropic functions of *Tgm4*. First, *Tgm4* expression has so far only been detected in the prostate [58]–[60], and never in any other tissues of a male or a female [61], [62], thus it should only affect ejaculate composition. Second, the only annotated domains in the Tgm4 protein are related to the formation of γ-glutamyl-ε-lysine bridges in its target proteins (www.ensembl.org), suggesting that it has a limited biological role. Third, although transglutaminases may alter the sperm surface *in vitro* [63], [64], Tgm4 has never been detected on the sperm surface [65], [66], suggesting it does not directly affect the gamete. Fourth, *Tgm4* has accumulated multiple loss-of-function mutations in some species that do not form a plug [67], which is not predicted if Tgm4 functions outside the context of plug formation.

Although the present study demonstrates the importance of the copulatory plug in non-competitive matings, it does not reject the hypothesis that the copulatory plug evolved in response to sperm competition [20], which occurs when a female mates with more than one male during a single fertile period [68]. Copulatory plugs are larger and show stronger coagulation intensity in species with high levels of inferred sperm competition [21], [22], [69], and have been lost in some species that experience low levels of sperm competition [67], [70], [71]. Some copulatory plug proteins evolve rapidly in species with high levels of inferred sperm competition, which is predicted if the plug inhibits female remating [67], [70]–[73]. In mice, the copulatory plug forms immediately upon ejaculation and remains intact for approximately 24 hours [20 and unpublished data], which is longer than the 4–12 hours that a female is able to be fertilized during her estrus cycle. Males contribute protease inhibitors in their ejaculates, which may function to preserve their copulatory plugs from female degradation [33]. Interestingly, males missing one of these protease inhibitors make a plug that degrades more quickly than wild type, which is associated with fertility defects [74]. Although the above patterns suggest the plug inhibits female remating, over 20% of wild caught pregnant females carry a litter sired by more than one male [75], [76], suggesting the plug is an imperfect barrier, and females or competitor males sometimes remove the plug [77]–[81].

Interestingly, copulatory plugs do not always bias fertilizations towards the first male to mate in one-female-two-male mating experiments [82]–[85], and some evolutionary patterns do not fit the sperm competition hypothesis. For example, the socially and genetically monogamous rodent *Peromyscus polionotus* forms a plug [86], [87]. By showing that the copulatory plug is correlated with normal fertility in one-male-one-female matings, the current study offers an explanation for the evolutionary maintenance of the copulatory plug in the absence of intense sperm competition. For example, the copulatory plug may prevent loss of semen [52], promote transport of semen through the female's reproductive tract [16], [41], [42], [53], [54], contribute to the threshold stimulation females require for proper implantation and pregnancy [55], and/or serve as a reservoir for the slow release of sperm in the female reproductive tract [88]. In reality, the copulatory plug may have multiple functions and the genetic model presented here enables unprecedented power to begin dissecting these hypotheses.

Many human seminal fluid proteins have orthologs in mouse ejaculates, including Tgm4 [33]. Even though human ejaculates do not form copulatory plugs, human seminal fluid enters a phase of coagulation and liquefaction [89], and defects in these transitions have been associated with subfertility [90]. There are 250 known nucleotide polymorphisms in human *Tgm4* mRNA, including 120 missense mutations (www.ensembl.org version 69), and Tgm4 was not detected in all ejaculates of five humans [91]. Future studies may reveal genetic and proteomic variation in *Tgm4* associated with differences in human male fertility.

## Materials and Methods

### Ethics statement

All mouse husbandry techniques, experimental methods, and personnel involved were approved by the University of Southern California's Institute for Animal Care and Use Committee, protocols #11394 and #11777.

### *Tgm4* “knockout first” allele

The *Tgm4* knockout mouse model was constructed by the multi-institutional Knockout Mouse Project [50], [51]. A ∼7 kb “knockout first” cassette was inserted into the C57BL/6N (6N) genetic background (project #CSD30105). Alternative crossing to Cre and/or FLP mice allows for further genetic modification of the knockout allele, but was unnecessary in the present study.

### Mouse husbandry

All experimental males used in this study had 6N parents that were heterozygous for the knockout (KO) and wild type (+) allele. When possible, all three genotypes were taken from the same litter to control for simple maternal effects.

Sires and dams were paired for one to two weeks, then separated so the dam gave birth in isolation. Between 21–28 days after birth, males were weaned in groups until genotyping, at which point they were separated into their own cages to avoid dominance interactions between brothers. Sexually mature males show reduced fertility when grouped together, presumably as a result of dominance interactions [92]. Females were weaned in groups of up to three individuals. All three possible male 6N genotypes - but only homozygous wild type 6N females - were used in various experiments described below.

Shortly after weaning, ear snips were taken for PCR-based genotyping. DNA isolated from ear snips was genotyped with four PCR reactions. Two PCR reactions specifically amplified the wild type allele: Reaction 1 primers (5′-AGGTGAAAAACCAAGAAATACCATC-3′ and 5′-CTATTCCAAAACCACCAGACAGTAC-3′) amplified a 704 bp fragment and Reaction 2 primers (GTGGACAGATATTCACTCTGAAGGT and GGAAACACCAATAGAAAAGTGAGTC) amplified a 1,170 bp fragment. Two PCR reactions specifically amplified the knockout first allele: Reaction 3 primers (GCTTTACATGTGTTTAGTCGAGGTT and GTTAAAGTTGTTCTGCTTCATCAGC) amplified a 1,244 bp fragment and Reaction 4 primers (GATTAAATATGATGAAAACGGCAAC and ATTATTTTTGACACCAGACCAACTG) amplified a 1,349 bp fragment. DNA was amplified using 35 cycles of denaturation (94 C, 20 seconds), annealing (58 C, 20 seconds) and extension (70 C and 40 seconds for Reaction 1, 70 C and 80 seconds for the other three reactions). All PCR reactions used Fermentas 2× PCR premix. Presence/absence of bands was scored on agarose gels. Only genotypes consistent across all four reactions were included in experiments.

### Male reproductive phenotypes, *in vivo*

All experimental males were individually paired with homozygous wild type 6N females. Males were between 60 and 90 days old. For the 3-hour and 20-hour assays (see below), ∼28 day-old females were induced to ovulate using standard techniques [93], [94]. Briefly, females were administered 5U Pregnant Mare's Serum Gonadotropin (PMSG) followed 48 hours later by 5U Human Chorionic Gonadotropin (hCG). For the 10–14 day assays (see below), females between 2 and 10 months old were used and ovulation was not artificially induced.

#### 3–hour assays

Twelve hours after administration of hCG, males and females were paired, left together for three hours, and then females were removed and sacrificed. Female reproductive tracts, extending from the vagina through the cervix and uterine horns, were dissected and examined under a dissecting microscope to assess i) the presence/absence of sperm, ii) the presence/absence of a copulatory plug, and iii) the number of oocytes with two pronuclei, a sign of recent fertilization [following 94]. Two different researchers who were blind to genotype measured uterine horns digitally, from a subset of females. Since their measurements were significantly correlated (Pearson's product-moment correlation coefficient = 0.84, P = 10^−11^), mean values were used for subsequent statistical analyses.

#### 20–hour assays

Males and females were paired immediately after administration of hCG and females were removed 20 hours later and the same phenotypes were gathered as for the 3-hour assays.

#### 10–14 day assays

Males and females were paired for 10 to 14 days, without hormonal induction of estrus, and then separated. Females were monitored for pregnancy and parturition, and litters were monitored to determine if they reached weaning age. All weanlings were sexed and counted. For a subset of randomly chosen litters, weanlings were weighed. To control for maternal experience, no pairings were included if they occurred prior to a female's first successful litter.

### Male reproductive phenotypes, *in vitro*

Between 2–6 months of age, a subset of experimental males were sacrificed, standard measurements taken, and testes and seminal vesicles dissected and weighed.

#### Sperm count and sperm motility

To determine sperm numbers and sperm motility, one caudal epididymis was placed in 100 µl pre-warmed Dulbecco's and minced with 27G needles. The minced epididymis was placed at 37 degrees C for one hour to allow sperm to swim free of cellular debris. The medium was swirled once, cellular debris allowed to settle, and 5 µl of this mixture placed on a Makler Chamber for quantification of sperm motility [95], [96]. Sperm motility was quantified as the average number of sperm that entered a 0.1 µl cell in a Makler Chamber per second. A total of 10 cells were observed for 10 seconds each. An additional 5 µl of heat-shocked sperm suspensions was used to quantify sperm count on a Makler Chamber.

### Statistical analyses

Unless otherwise stated, Student's *t*-tests were used to compare phenotypes among groups. In all *t*-tests, assumptions of normality and equal variances were confirmed using Shapiro-Wilk tests and F-tests, respectively. In a few comparisons indicated above, the two groups being compared had significantly different variances; in these cases Welch's *t*-test [97] was used. Importantly, no conclusions changed if Student's *t*-tests, Welch's *t*-test, or non-parametric Mann-Whitney *U* tests were used in any comparisons.

Testis and seminal vesicle weight were each analyzed in a full factorial ANCOVA using male genotype (knockout vs. wild type) and body weight as factors. An ANCOVA was employed to account for the potential covariation of testis or seminal vesicle weight with body weight. To test for differences in the number of litters born to *Tgm4* knockout vs. wild type males, a 2×2 contingency table was tested against a χ^2^ distribution. All statistical analyses were performed in R (www.r-project.org) or customized Python scripts (www.python.org).
